# Supplementary material for: Crickets evade bats via olfaction beyond acoustic cues
Source: eLife. 2026 Aug 21;15:RP110936. doi: 10.7554/eLife.110936 (PMC13498323; doi:10.7554/eLife.110936)
Supplement: Supplementary file 3 [file elife-110936-fig2-data5.zip › compounds 3.pdf]

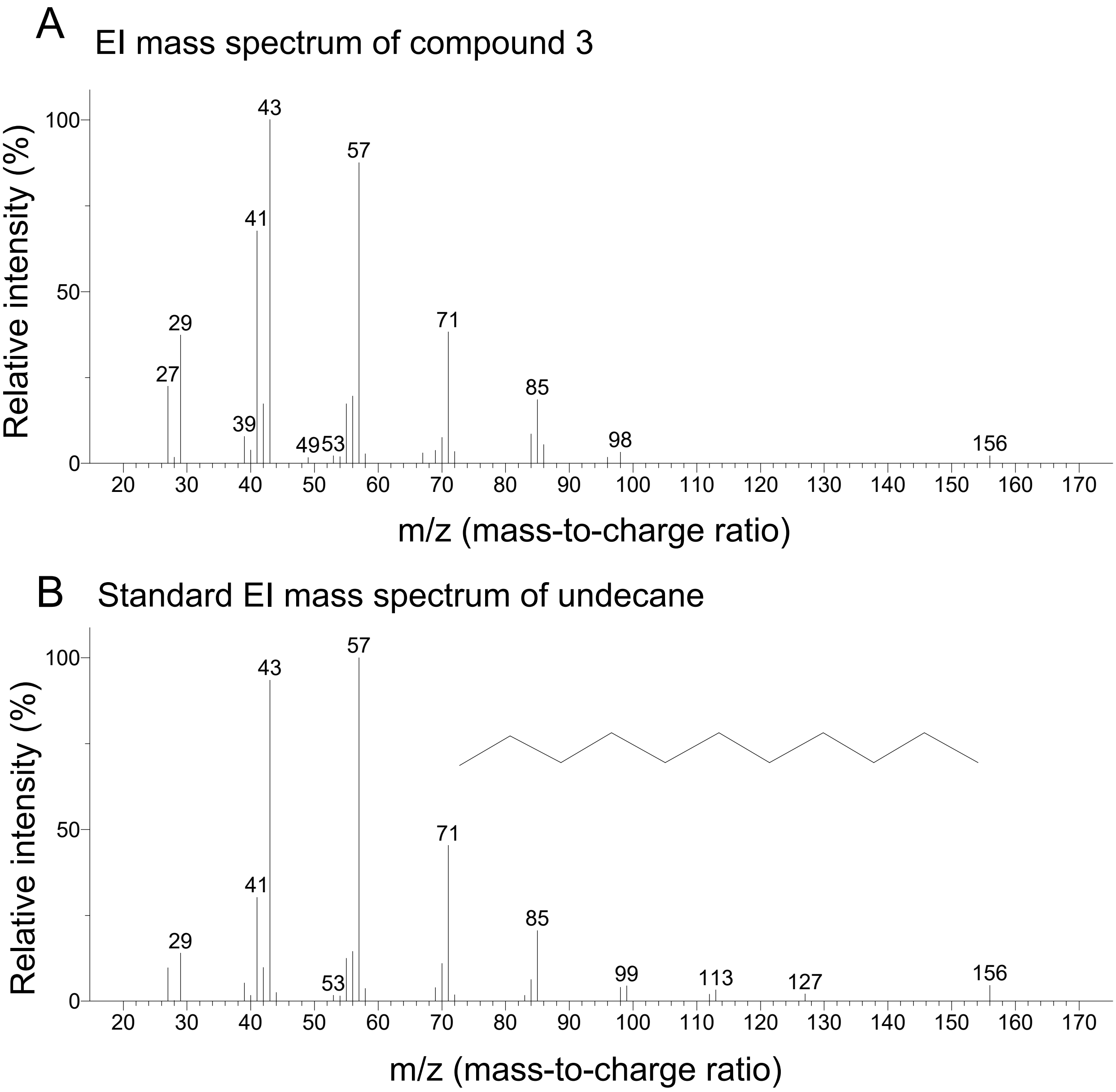
A
EI mass spectrum of compound 3
Relative intensity (%)
m/z (mass-to-charge ratio)
27
29
39
41
43
49
53
57
71
85
98
156
B
Standard EI mass spectrum of undecane
Relative intensity (%)
m/z (mass-to-charge ratio)
29
41
43
53
57
71
85
99
113
127
156
