# Supplementary material for: Crickets evade bats via olfaction beyond acoustic cues
Source: eLife. 2026 Aug 21;15:RP110936. doi: 10.7554/eLife.110936 (PMC13498323; doi:10.7554/eLife.110936)
Supplement: Supplementary file 3 [file elife-110936-fig2-data5.zip › compounds 4.pdf]

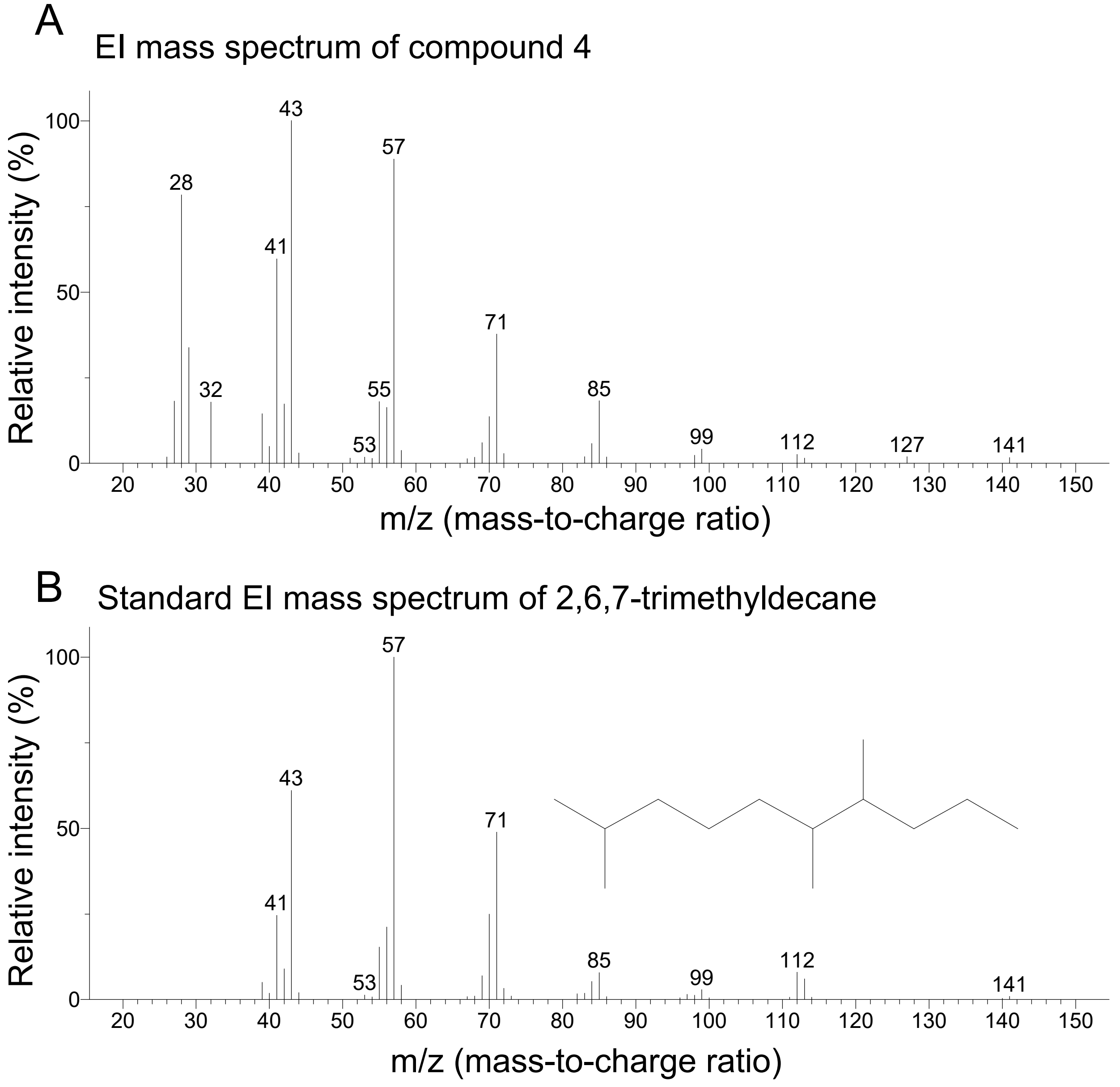
A
EI mass spectrum of compound 4
Relative intensity (%)
m/z (mass-to-charge ratio)
28
32
41
43
53
55
57
71
85
99
112
127
141
B
Standard EI mass spectrum of 2,6,7-trimethyldecane
Relative intensity (%)
m/z (mass-to-charge ratio)
41
43
53
57
71
85
99
112
141
