# Supplementary material for: Crickets evade bats via olfaction beyond acoustic cues
Source: eLife. 2026 Aug 21;15:RP110936. doi: 10.7554/eLife.110936 (PMC13498323; doi:10.7554/eLife.110936)
Supplement: Supplementary file 3 [file elife-110936-fig2-data5.zip › compounds 5.pdf]

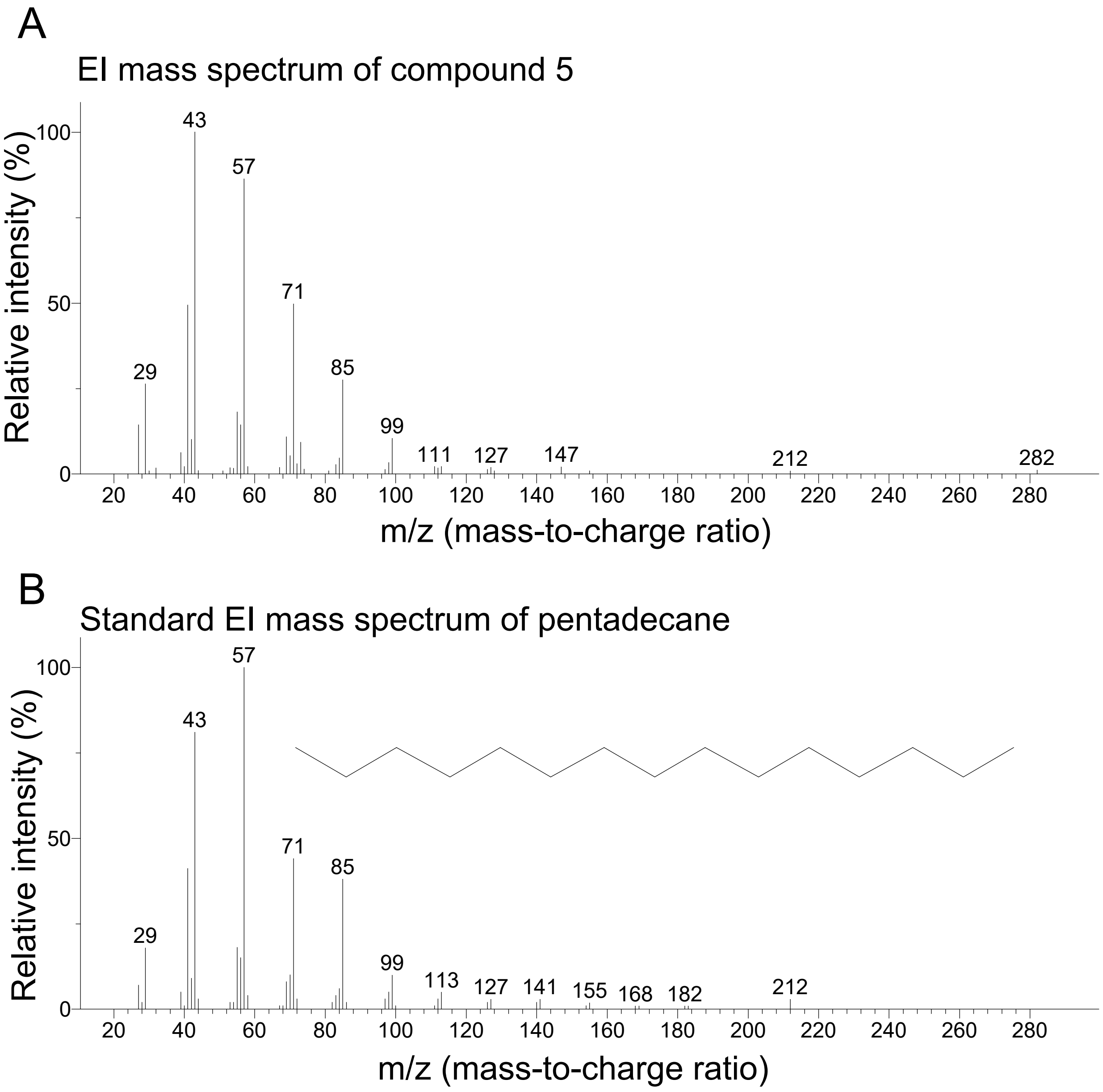
A
EI mass spectrum of compound 5
Relative intensity (%)
0
50
100
29
43
57
71
85
99
111
127
147
212
282
20
40
60
80
100
120
140
160
180
200
220
240
260
280
m/z (mass-to-charge ratio)
B
Standard EI mass spectrum of pentadecane
Relative intensity (%)
0
50
100
29
43
57
71
85
99
113
127
141
155
168
182
212
20
40
60
80
100
120
140
160
180
200
220
240
260
280
m/z (mass-to-charge ratio)
