# Supplementary material for: Crickets evade bats via olfaction beyond acoustic cues
Source: eLife. 2026 Aug 21;15:RP110936. doi: 10.7554/eLife.110936 (PMC13498323; doi:10.7554/eLife.110936)
Supplement: Supplementary file 3 [file elife-110936-fig2-data5.zip › compounds 6.pdf]

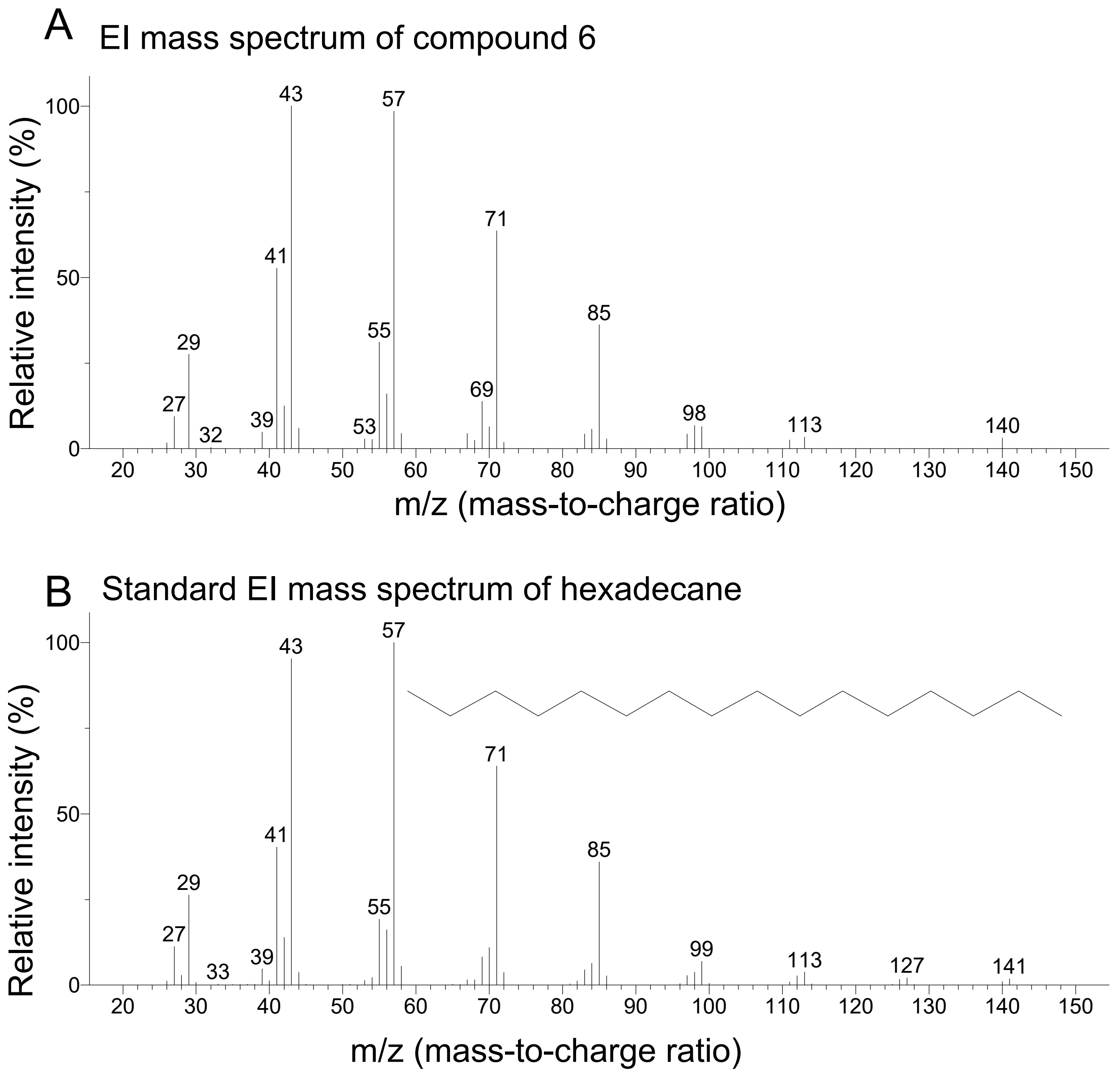
A
EI mass spectrum of compound 6
Relative intensity (%)
0
50
100
27
29
32
39
41
43
53
55
57
69
71
85
98
113
140
20 30 40 50 60 70 80 90 100 110 120 130 140 150
m/z (mass-to-charge ratio)
B
Standard EI mass spectrum of hexadecane
Relative intensity (%)
0
50
100
27
29
33
39
41
43
55
57
71
85
99
113
127
141
20 30 40 50 60 70 80 90 100 110 120 130 140 150
m/z (mass-to-charge ratio)
