# Supplementary material for: Crickets evade bats via olfaction beyond acoustic cues
Source: eLife. 2026 Aug 21;15:RP110936. doi: 10.7554/eLife.110936 (PMC13498323; doi:10.7554/eLife.110936)
Supplement: Supplementary file 3 [file elife-110936-fig2-data5.zip › compounds 1.pdf]

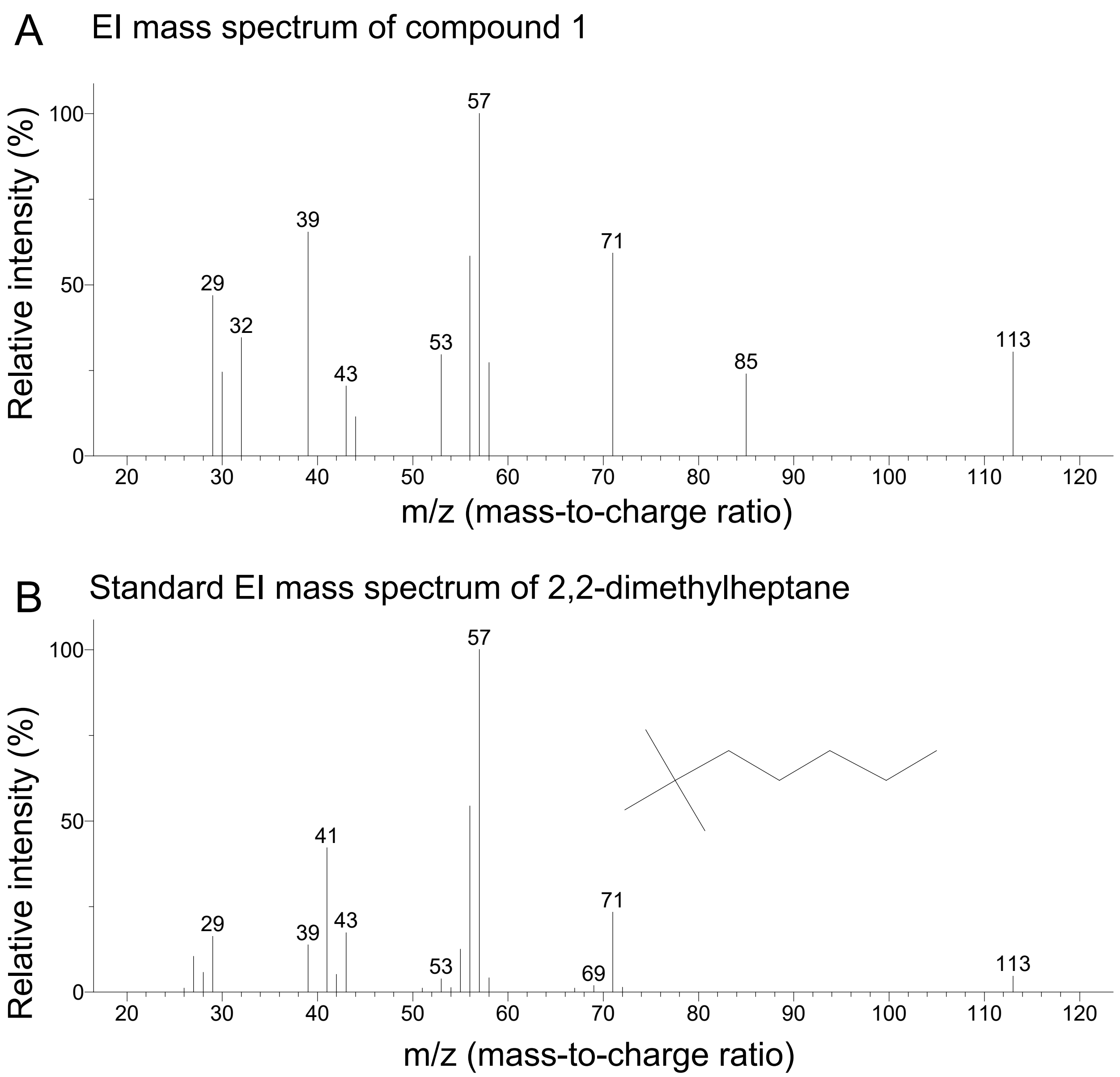
A
EI mass spectrum of compound 1
Relative intensity (%)
0
50
100
29
32
39
43
53
57
71
85
113
20
30
40
50
60
70
80
90
100
110
120
m/z (mass-to-charge ratio)
B
Standard EI mass spectrum of 2,2-dimethylheptane
Relative intensity (%)
0
50
100
29
39
41
43
53
57
69
71
113
20
30
40
50
60
70
80
90
100
110
120
m/z (mass-to-charge ratio)
