# Supplementary material for: Crickets evade bats via olfaction beyond acoustic cues
Source: eLife. 2026 Aug 21;15:RP110936. doi: 10.7554/eLife.110936 (PMC13498323; doi:10.7554/eLife.110936)
Supplement: Supplementary file 3 [file elife-110936-fig2-data5.zip › compounds 2.pdf]

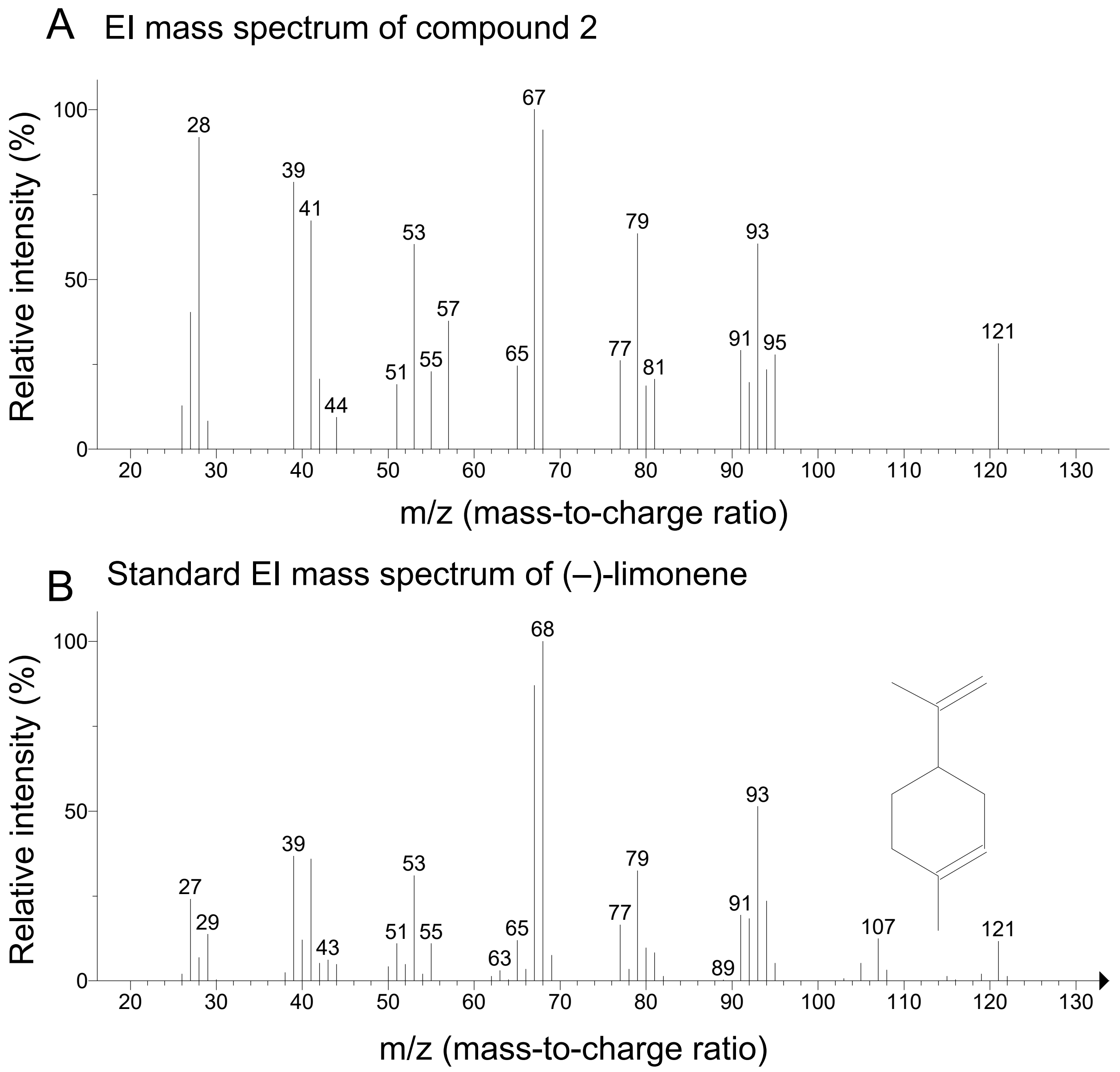
A
EI mass spectrum of compound 2
Relative intensity (%)
0
50
100
28
39
41
44
51
53
55
57
65
67
77
79
81
91
93
95
121
20
30
40
50
60
70
80
90
100
110
120
130
m/z (mass-to-charge ratio)
B
Standard EI mass spectrum of (–)-limonene
Relative intensity (%)
0
50
100
27
29
39
43
51
53
55
63
65
68
77
79
89
91
93
107
121
20
30
40
50
60
70
80
90
100
110
120
130
m/z (mass-to-charge ratio)
