# Supplementary material for: Crickets evade bats via olfaction beyond acoustic cues
Source: eLife. 2026 Aug 21;15:RP110936. doi: 10.7554/eLife.110936 (PMC13498323; doi:10.7554/eLife.110936)
Supplement: Peer review history

# Editor report: eLife Assessment

**DOI:** 10.7554/eLife.110936.3.sa0

**Authors:** Sergio Rasmann

**Affiliations:** University of Neuchâtel Switzerland

**Keywords:** Convincing, Important

This study provides **important** evidence that crickets use bat-associated odors as cues of predation risk, extending the classic bat-insect arms race beyond its traditional acoustic framework. The authors combine fecal metabarcoding, behavioral assays, electrophysiology, chemical analyses, and field observations to show that *Loxoblemmus equestris* avoids the odor of the insectivorous bat *Scotophilus kuhlii*, and that synthetic limonene can elicit antennal responses, avoidance in the laboratory, and reduced calling activity in the field. Together, the multidisciplinary results provide a coherent and **convincing** chain of evidence for an olfactory component of predator detection in this bat-insect system. The work will be of broad interest to researchers in sensory ecology, chemical ecology, predator-prey interactions, and bat-insect coevolution.

# Referee report: Reviewer #1 (Public review)

**DOI:** 10.7554/eLife.110936.3.sa1

[Editors' note: this version has been assessed by the Reviewing Editor without further input from the original reviewers. The authors have addressed the comments raised in the previous round of review.]

The manuscript examines whether insects can use bat odor as a cue of predation risk. The authors focus on the insectivorous bat Scotophilus kuhlii and the cricket Loxoblemmus equestris. They first use fecal DNA metabarcoding to show that crickets are part of the bat's diet, and field surveys to show that L. equestris is abundant at local foraging sites. In laboratory Y-tube assays, the authors show that crickets strongly avoid air carrying bat body odor. Gas chromatography coupled with electroantennographic detection showed that cricket antennae respond to components of bat odor. Chemical analyses identified several volatile compounds, with 2,2-dimethylheptane and (−)-limonene associated with antennal responses. Further analyses suggested that snout secretions are likely to contribute to the bat's body odor. The authors then tested individual compounds. Among the commercially available candidates, (−)-limonene elicited a strong antennal response and was sufficient to cause avoidance in the olfactometer. In field plots, spraying (−)-limonene reduced cricket calling activity relative to pre-exposure levels, whereas calling increased in control plots treated with hexane. Overall, the study argues that crickets can detect a vertebrate predator through olfactory cues and that a single bat-associated volatile can trigger antipredator behavior.

This is an interesting and enjoyable study that addresses an understudied aspect of predator-prey interactions. The manuscript is clearly written, the experiments are presented in a logical sequence, and the figures are crisp and easy to follow. I really appreciated the combination of behavioral assays, electrophysiology, chemical analysis, and field observations.

# Referee report: Reviewer #2 (Public review)

**DOI:** 10.7554/eLife.110936.3.sa2

Many insects possess extremely sensitive olfactory systems that can detect chemical signals from distances of several kilometers. For decades, the arms race between bats and insects has served as a prime example of acoustic co-evolution. The auditory adaptations of insects to echolocation have been well documented. Cricket has a multi-sensory predator recognition system with keen olfactory, tactile, and auditory senses. However, whether crickets can use the scent of bats to avoid them remains unknown at present. The authors hypothesized that cricket prey (Loxoblemmus equestris) might eavesdrop on predator bat (Scotophilus kuhlii) VOCs as an early warning. L. equestris is one of the prey species of S. kuhlii, and the authors demonstrated that the body odor of the insectivorous bat S. kuhlii triggers robust avoidance and electrophysiological responses in the cricket L. equestris, and that a single compound, (-)-limonene, is sufficient to elicit this avoidance in the laboratory and suppress calling in the field. Overall, this paper has a complete chain of evidence and should be a highly praised study.

# Author comment: Author response

**DOI:** 10.7554/eLife.110936.3.sa3

**Authors:** Yannan Li, Wenhao Zhang, Jiaqi Wei, Hanhong Xu, Jiang Feng, Aiqing Lin

**Affiliations:** Northeast Normal University Changchun China; Northeast Normal University Changchun China; South China Agricultural University Guangzhou China; South China Agricultural University Guangzhou China; Northeast Normal University Changchun China; Northeast Normal University Changchun China

The following is the authors’ response to the original reviews.

We sincerely thank you and the reviewers for the thoughtful evaluation of our manuscript and for the constructive comments and important suggestions. We are encouraged by the recognition that the study is valuable and of interest to researchers in sensory ecology, chemical ecology, predator-prey interactions, and bat-insect coevolution. We are also grateful that the reviewers acknowledged the integrative approach of our work, including fecal metabarcoding, behavioral assays, electrophysiological recordings, chemical analyses, and field observations.

We have carefully considered all comments and have revised the manuscript accordingly. In particular, we have made the following major revisions:

(1) We clarified the biological origin of limonene and expanded the discussion of possible bat-associated sources, including snout secretions and microbial contributions, while avoiding overinterpretation of limonene as an exclusively endogenous mammalian compound.

(2) We added more detailed descriptions of contamination controls, including instrument cleaning procedures, materials used for housing and handling, and blank-control results, to address the concern that limonene could have originated from human-associated or environmental contamination.

(3) We added individual-level odor data and revised the presentation of terpenoid profiles to show more clearly where limonene was detected and how its relative contribution varied among samples.

(4) We clarified the rationale for the concentration choices used in the electrophysiological and field experiments, emphasizing that these assays were designed to test physiological detectability and functional sufficiency rather than to reproduce exact natural emission concentrations or determine response thresholds.

(5) We revised our interpretation of limonene more cautiously. We now state that limonene is sufficient to trigger avoidance responses, while we acknowledge that its natural ecological specificity, concentration dynamics, and interactions with other bat odor components require further investigation.

(6) We corrected the terminology related to limonene enantiomers. Because our GC-MS and GC-EAD analyses did not use an enantioselective column, we replaced “(-)-limonene” with “limonene” throughout the manuscript, figures, legends, and supplementary materials.

(7) We improved the figures and supporting materials by moving the figure illustrating predator-prey relationship into the main text, adding electrophysiological traces from all tested crickets, revising figure legends, clarifying the rationale for comparing bat body odor with air controls, and providing additional chemical-identification details in the supplementary materials.

(8) We checked and clarified statistical annotations, including the exact adjusted P-values for relevant comparisons.

We believe these revisions have substantially improved the clarity, rigor, and balance of the manuscript. We hope that the revised manuscript and the detailed point-by-point responses satisfactorily address all concerns raised.

We are confident that our study represents an important contribution, as it fundamentally expands the traditional acoustic-centred view of bat–insect interactions by demonstrating that crickets can use olfaction as a complementary sensory modality to detect bat odors and initiate avoidance behavior. The multidisciplinary evidence, integrating behavioral assays, electrophysiology, chemical profiling, and field validation, provides convincing support for this novel olfactory pathway.

**eLife Assessment**

This valuable study raises the intriguing possibility that crickets use bat-associated odors as cues of predation risk, extending the classic bat-insect arms race beyond its usual acoustic framework. The authors combine fecal metabarcoding, behavioral assays, electrophysiology, chemical analyses, and field observations to show that *Loxoblemmus equestris* avoids the odor of the insectivorous bat *Scotophilus kuhlii*, and that synthetic (-)-limonene can elicit antennal responses, avoidance in the laboratory, and reduced calling activity in the field. However, the evidence is currently incomplete because the identity, biological source, natural concentration, and ecological specificity of limonene as a bat-derived predator cue require stronger support, including clearer quantification, contamination controls, individual-level odor data, and evidence that crickets can distinguish bat-associated limonene from common environmental sources. The work will be of interest to researchers in sensory ecology, chemical ecology, predator-prey interactions, and bat-insect coevolution.

We sincerely thank the editors for the positive and constructive assessment of our work. We greatly appreciate the recognition of our study’s value and its potential interest to multiple research communities.

We have carefully considered all comments and have revised the manuscript accordingly. The revisions include clarifications of limonene’s biological origin and ecological specificity, strengthened contamination controls and individual-level odor data, clearer rationales for experimental concentration choices, and more cautious interpretation throughout the manuscript. All changes are addressed in detail in our point-by-point responses below.

Importantly, the central conclusion of our study—that crickets can detect and avoid bat odor through olfaction, and that limonene is sufficient to trigger avoidance responses in both laboratory and field settings—remains robustly supported by the multidisciplinary evidence we present. We believe the revised manuscript now provides a clearer, more balanced, and scientifically rigorous account of our findings, and we hope it meets the standards of eLife.

**Public Reviews:**

**Reviewer #1 (Public review):**

The manuscript examines whether insects can use bat odor as a cue of predation risk. The authors focus on the insectivorous bat *Scotophilus kuhlii* and the cricket *Loxoblemmus equestris*. They first use fecal DNA metabarcoding to show that crickets are part of the bat's diet, and field surveys to show that *L. equestris* is abundant at local foraging sites. In laboratory Y-tube assays, the authors show that crickets strongly avoid air carrying bat body odor. Gas chromatography coupled with electroantennographic detection showed that cricket antennae respond to components of bat odor. Chemical analyses identified several volatile compounds, with 2,2-dimethylheptane and (−)-limonene associated with antennal responses. Further analyses suggested that snout secretions are likely to contribute to the bat's body odor. The authors then tested individual compounds. Among the commercially available candidates, (−)-limonene elicited a strong antennal response and was sufficient to cause avoidance in the olfactometer. In field plots, spraying (−)-limonene reduced cricket calling activity relative to pre-exposure levels, whereas calling increased in control plots treated with hexane. Overall, the study argues that crickets can detect a vertebrate predator through olfactory cues and that a single bat-associated volatile can trigger antipredator behavior.

This is an interesting and enjoyable study that addresses an understudied aspect of predator-prey interactions. The manuscript is clearly written, the experiments are presented in a logical sequence, and the figures are crisp and easy to follow. I really appreciated the combination of behavioral assays, electrophysiology, chemical analysis, and field observations.

We sincerely thank you for your very positive and encouraging evaluation of our work. We are delighted that you found the study interesting and enjoyable. We also appreciate your kind remarks about the clarity of the manuscript, the logical flow of the experiments, and the quality of the figures.

We are especially grateful that you recognized the value of our integrative approach. Combining behavioral assays, electrophysiology, chemical analysis, and field observations was central to our study design, and we are pleased that this approach resonated with you.

You provided an accurate and comprehensive summary of our work. You confirmed that our main narrative is clear and logically coherent. Specifically, you followed our progression from establishing the predator–prey relationship, to demonstrating olfactory avoidance, to identifying limonene as an active compound, and finally to validating its behavioral effects in both laboratory and field settings.

We have carefully considered all your constructive comments and suggestions. We address them in detail in our point-by-point responses below. Your feedback has been extremely helpful, and we believe the revised manuscript is substantially stronger as a result.

My main issue concerns the identity and biological origin of the proposed bat odor cue, (−)-limonene. Limonene seems like an unusual compound to be emitted endogenously by a mammal, particularly by an insectivorous bat. It would be helpful if the authors could clarify whether mammals are known to synthesize this compound de novo, and, if not, what the likely source of this plant-associated terpene would be in *S. kuhlii*. Possible sources could include environmental exposure, diet, roosting material, handling, or temporary housing conditions.

I do not doubt that crickets avoid synthetic (−)-limonene. Indeed, this result is quite plausible given that limonene is widely used in insect repellent or repellent-associated fragrance products. However, this also makes contamination an important issue to address explicitly. How did the authors exclude the possibility that limonene entered the samples from human-associated sources, such as insect repellents, soaps, cleaning products, field equipment, cloth bags, cages, gloves, or other materials used while handling wild-caught bats? It would strengthen the manuscript to report limonene levels for individual bat odor collections, all relevant blanks, and any handling or housing controls.

More broadly, given the common occurrence of limonene in plants and human-associated products, I am not yet convinced that it would function as a reliable "keystone kairomone" as suggested around line 253. How would crickets distinguish bat-associated limonene from limonene emitted by a mint leaf, citrus peel, pine material, or other non-threatening environmental sources? The authors may wish to soften this interpretation or provide additional evidence that crickets respond to limonene in a bat-specific context, perhaps through concentration, temporal patterning, co-occurring volatiles, or enantiomeric composition.

We sincerely thank you for your critical and constructive comments. Your questions regarding the identity, biological origin, and ecological specificity of limonene are insightful and have helped us substantially strengthen the manuscript. We address each of your points below.

On the biological origin of limonene and whether mammals synthesize it *de novo*

You raised an important point that limonene seems unusual for a mammal to emit endogenously. We fully agree. We agree that direct evidence for *de novo* synthesis of limonene in mammals is currently limited.

However, limonene in bat body odor could still have biological origins. First, it may originate from skin- or gland-associated microbiota. Recent work has shown that skin-associated microorganisms can substantially shape bat volatile odor profiles (Sun et al., 2026, BMC Biology), and some microbes possess enzymes capable of terpene biosynthesis. Second, previous studies have independently reported limonene in the secretions of several bat species (Faulkes et al., 2019, PeerJ; Zhang et al., 2022, Ann. N.Y. Acad. Sci.). This suggests that its presence in bats is not unique to our study. Third, our own analyses detected limonene in hair and snout secretions, but not in faeces or blank controls. This pattern is consistent with a biological source associated with the body surface, rather than diet or environmental deposition.

We have now expanded our Discussion to cover these possibilities more explicitly. We also emphasize that the exact source, i.e., endogenous, microbial, or otherwise, remains an open question that warrants future investigation. Please see lines 258–273 of the clean version of the revised manuscript, or see the excerpt below:

“Although limonene reliably induced avoidance behaviour in crickets, two related questions still merit careful consideration. One question is whether the limonene we identified genuinely originates from bats or reflects contamination during sampling. Limonene is common in plants and numerous consumer products (Boncan et al., 2020; Schuman, 2023), making its endogenous production by a mammal seem unusual. Nevertheless, multiple lines of evidence militate against contamination. First, we adhered to rigorous protocols. For example, all instruments were cleaned with ethanol and oven-dried before each use; bats were housed in stainless-steel cages, and cloth bags had been rinsed with purified water. Second, limonene was absent from all blank controls, including empty-chamber air samples and clean swabs, and was not detected in bat faecal samples. In contrast, it was consistently identified in hair and snout-secretion samples from bats. Third, independent studies have similarly identified limonene in the secretions of other bat species (Faulkes et al., 2019; Zhang et al., 2022). Furthermore, emerging evidence indicates skin-associated microbes may contribute to bat volatile profiles, with some taxa possessing enzymes involved in terpene biosynthesis (Sun et al., 2026). Taken together, these observations point towards an endogenous or microbe-mediated source, although the exact biosynthetic pathway remains to be determined.”

On contamination from human-associated sources

You asked how we excluded the possibility that limonene entered our samples through handling, equipment, cleaning products, or other human-associated sources. We appreciate this concern and have addressed it in detail.

We believe contamination is highly unlikely for several reasons. First, we followed strict protocols throughout. All instruments were cleaned with ethanol and oven-dried before and after each use. We used stainless-steel cages and cloth bags made of degreased bleached cotton washed with purified water. These materials are not sources of terpenes. Second, we ran multiple blanks. Limonene was not detected in any empty-chamber air controls or in blank cotton swabs. In contrast, it was consistently found in multiple bat snout-secretion samples. This clear difference between samples and blanks strongly argues against contamination. Third, we now report individual-level odor data (see new Figure 3; Supplementary Table 5.xlsx). These data show that limonene was consistently present across individual bats. It did not appear sporadically, as one would expect from accidental contamination.

In the revised manuscript, we have added detailed descriptions of our contamination controls in the Methods section (Please see lines 405–407, 443–444, 471–477 of the clean version of the revised manuscript, or see the excerpt below). We have also included the blank-control results (Supplementary Table 5.xlsx), as you suggested.

Lines 405–407: “Prior to sampling, all glassware was thoroughly rinsed with ethanol and dried in an oven at 120°C, and volatile odor collection was conducted in a dedicated odor-free room to minimize environmental contamination.”

Lines 443–444: “The empty-chamber controls were used to account for potential background signals from the experimental system and to provide a baseline for comparison with bat odor extracts.”

Lines 471–477: “Upon capture, bats were placed in clean stainless-steel cages and kept in groups consistent with their natural social associations during the brief interval prior to immediate odor sampling. Hair samples (10 mg per individual) were clipped from dorsal and ventral regions. Snout secretions were collected using sterile cotton swabs (CS15-005, Shenzhen SihuaBo Technology Co., Ltd., China), with two blank swabs as controls. These blank swab controls were included to account for potential volatile contamination from ambient air or the swab material itself (Supplementary Table 5).”

On how crickets distinguish bat-associated limonene from environmental sources

You raised a thoughtful question about ecological specificity. Given that limonene is abundant in mint, citrus peel, pine, and other non-threatening plants, how would crickets use it as a reliable indicator of bat presence?

We agree with you completely. We do not claim that limonene alone serves as an unambiguous bat-specific signal. Instead, our interpretation is more nuanced. We argue that elemental perception represents one effective strategy within a broader olfactory toolkit. It does not exclude the importance of other cues.

In our revised manuscript, we have softened our interpretation accordingly. We now state explicitly that limonene is sufficient to trigger avoidance under our experimental conditions, but we do not interpret it as a uniquely bat-specific keystone kairomone. We also discuss mechanisms that could help crickets reduce false alarms under natural conditions. These include concentration differences, temporal patterning (bats are active at night), spatial context (specific foraging habitats), co-occurrence with other bat-specific volatiles, and possibly enantiomeric composition. Please see lines 274–292 of the clean version of the revised manuscript, or see the excerpt below.

Lines 274–292: “The second question is how crickets might distinguish bat-derived limonene from environmental sources of this compound, given its prevalence in mint, citrus peel, pine and other non-threatening plants (Boncan et al., 2020; Schuman, 2023). It seems implausible that crickets could simply rely on limonene per se to differentiate a bat from a leaf. Two non-exclusive mechanisms could help resolve this issue. First, limonene need not be the only olfactory cue mediating risk perception. Our findings establish the sufficiency of limonene as an avoidance trigger, but do not preclude a role for other odor components. The crickets’ antennal responses to other bat volatiles in our GC–EAD analyses suggest more complex peripheral perception. Additional compounds, either alone or in synergistic blends, may modulate the full behavioral response in nature. Therefore, elemental perception via limonene likely represents one effective strategy within a broader olfactory toolkit available to insects. Second, crickets may discriminate bat-derived limonene through context-specific cues (e.g., temporal and spatial patterning, co-occurrence with other bat-specific compounds) to minimize false alarms. Comparative studies on enantiomeric specificity and detection thresholds of cricket olfactory sensory neurons will be essential. Equally critical will be future efforts to quantify natural bat odor composition, limonene release rates, ambient exposure concentrations, and odor-plume dynamics, which together will inform ecologically valid stimulus design in controlled assays. Critically, our field data confirm that limonene exposure in nature robustly triggers an adaptive anti-predator response, irrespective of the precise discrimination mechanism.”

We acknowledge that fully testing these ideas would require substantial additional work. We have therefore framed this as an important direction for future research, rather than as a resolved issue in the present study.

We thank you again for these insightful comments. Your feedback has helped us present a more rigorous, balanced, and transparent account of our work.

**Reviewer #2 (Public review):**

Summary:

Many insects possess extremely sensitive olfactory systems that can detect chemical signals from distances of several kilometers. For decades, the arms race between bats and insects has served as a prime example of acoustic co-evolution. The auditory adaptations of insects to echolocation have been well documented. Cricket has a multi-sensory predator recognition system with keen olfactory, tactile, and auditory senses. However, whether crickets can use the scent of bats to avoid them remains unknown at present. The authors hypothesized that cricket prey (*Loxoblemmus equestris*) might eavesdrop on predator bat (*Scotophilus kuhlii*) VOCs as an early warning. *L. equestris* is one of the prey species of *S. kuhlii*, and the authors demonstrated that the body odor of the insectivorous bat *S. kuhlii* triggers robust avoidance and electrophysiological responses in the cricket *L. equestris*, and that a single compound, (-)-limonene, is sufficient to elicit this avoidance in the laboratory and suppress calling in the field. Overall, this paper has a complete chain of evidence and should be a highly praised study.

We sincerely thank you for your very positive and encouraging evaluation of our work. We are especially gratified that you recognized our study as having a "complete chain of evidence" and as a "highly praised study." This recognition means a great deal to us, given the multidisciplinary nature of our approach and the effort required to integrate behavioral, electrophysiological, chemical, and field data into a coherent narrative.

We also appreciate your accurate summary of our work. You correctly highlighted the broader context that while acoustic co-evolution between bats and insects is well documented, whether crickets can use bat scent as an early warning cue has remained unknown. Your summary confirms that our main findings are clear: the body odor of *S. kuhlii* triggers robust avoidance and electrophysiological responses in *L. equestris*, and that limonene alone is sufficient to elicit avoidance in the laboratory and suppress calling in the field.

We are particularly grateful that you acknowledged the multi-sensory nature of cricket predator recognition, with keen olfactory, tactile, and auditory senses. We agree that crickets are an excellent model for studying multimodal predator detection, and we hope our study encourages further exploration of olfaction in this classic predator–prey system.

We have carefully considered all your specific comments and suggestions. These include questions about the novelty framing of olfactory eavesdropping, the rationale for our concentration choices, and the presentation of electrophysiological comparisons. We address each of these points in detail in our point-by-point responses below. Your thoughtful feedback has been extremely helpful in improving the clarity and rigor of the manuscript.

Comments:

(1) Olfactory eavesdropping can transcend the evolutionary divide between vertebrate predators and invertebrate prey, enabling invertebrates to trigger defensive avoidance behaviors in response to predator-derived volatile odors. This phenomenon is empirically well-documented and requires no excessive emphasis.

Thank you for this comment. You are absolutely right that olfactory eavesdropping across the vertebrate–invertebrate divide is not a new concept in itself, and we acknowledge that this phenomenon has been well documented in previous studies.

However, we would like to clarify our intended emphasis. In the Introduction and Discussion, we have already stated that empirical examples combining chemical identification, electrophysiological validation, behavioral assays, and field confirmation within a direct predator–prey context remain relatively limited. This is especially true for the bat–insect system, where research has historically focused on acoustic interactions rather than olfaction.

We did not intend to overstate the novelty of olfactory eavesdropping per se. Instead, our emphasis was on providing a complete chain of evidence in a vertebrate–invertebrate predator–prey system that has traditionally been viewed through an acoustic lens. In that sense, we believe our study adds a complementary olfactory perspective to this classic system, rather than claiming to have discovered olfactory eavesdropping as a novel phenomenon. We hope this clarifies our position, as already stated in the original manuscript (lines 71–78, lines 88–90, lines 235–241 of the clean version of the revised manuscript, or see the excerpt below):

Lines 71–78: “However, a fundamental gap exists in understanding whether such olfactory eavesdropping can operate across the vast phylogenetic divide separating vertebrate predators and invertebrate prey (Apfelbach et al., 2015; Dicke and Grostal, 2001; Schoeppner and Relyea, 2005). Although olfactory interactions across broad taxonomic boundaries are widespread in nature, such as mosquitoes using host odors to blood-feed, elephants and moths sharing pheromonal components, and aroids chemically mimicking carrion to attract pollinating flies (Kang et al., 2023; Zaremska et al., 2022; Zhao et al., 2022), these interactions are primarily shaped by selective pressures tied to foraging, reproduction, or mutualisms, rather than by predation-related selection.”

Lines 88–90: “The bat–insect system presents an ideal model to address these questions. Despite the clear importance of olfaction to both taxa and its established role in predator–prey ecology, whether it plays any functional role in the iconic bat–insect arms race remains unexplored.”

Lines 235–241: “Beyond the specific bat–insect model, our work addresses a central question in sensory ecology: how chemical eavesdropping operates within predator–prey systems between phylogenetically distant taxa with fundamentally divergent olfactory systems (Adams et al., 2020; Emerson and Johnson, 2024; Kaupp, 2010). While intraphyletic kairomone detection is well-established (e.g., rodents avoiding carnivore odors, aphids responding to ladybug chemicals), compelling experimental evidence for such olfaction-mediated recognition across broad phylogenetic divides has been limited (Apfelbach et al., 2005; Ferrari et al., 2007; Tanis et al., 2018).”

(2) Without quantitative analysis and without knowing the relative content of this key substance limonene, I don't quite understand how to determine the concentration of limonene standard for EAD, as well as the concentration in field experiments. How is the concentration of limonene determined in field spraying, and is this actually the case in the wild environment?

Thank you for this question. We fully agree that knowing the natural concentrations and relative content of limonene in bat odor would be valuable. However, our experimental aims were not to mimic natural emission levels precisely. Instead, they were designed to answer two distinct questions: First, whether cricket antennae are physiologically capable of detecting limonene at all; and second, whether limonene alone is sufficient to trigger behavioral responses under controlled and semi-natural conditions.

For the EAG experiments, we selected a concentration gradient (0.001%, 0.01%, 0.1%, 1%, and 10% v/v) following standard practices in insect chemical ecology and referencing a previous study (Tang et al., 2024). The goal was to establish dose-dependent antennal sensitivity, not to match a specific natural concentration. Our data clearly show that cricket antennae respond across a range of concentrations, with stronger responses at higher doses.

For the field experiment, we used a 10% v/v limonene spray over 25 m^2^ plots. We acknowledge that this concentration does not quantitatively reflect natural bat emissions. Natural odor plumes are highly dynamic and depend on airflow, turbulence, temperature, humidity, vegetation structure, and distance from the source. Accurately reconstructing these natural dynamics would require detailed quantitative measurements of bat odor release rates and plume modeling, which were beyond the scope of the present study. Instead, our field experiment was designed for a functional purpose: to test whether limonene could alter cricket calling behavior under semi-natural conditions, using a concentration sufficient to produce a detectable odor stimulus in the field.

We also note that the field-applied concentration is comparable to what has been used in other chemical ecology studies testing the behavioral effects of single volatile compounds under natural or semi-natural conditions. In that context, our positive result supports the ecological relevance of limonene as an avoidance cue, without requiring that the exact applied concentration matches natural bat emissions.

We agree that quantitative characterization of natural bat odor composition, limonene release rates, and ambient exposure concentrations is an important direction for future research. According to your comments, we have added this point to the revised Discussion as a clear future direction (please see lines 286–290 of the clean version of the revised manuscript, or see the excerpt below). We have also clarified in the Methods that our assays were designed to test physiological detectability and functional sufficiency, rather than to establish concentration thresholds or mimic natural emissions exactly (please see lines 515–522, 556–558 of the clean version of the revised manuscript, or see the excerpt below).

Lines 286–290: “Comparative studies on enantiomeric specificity and detection thresholds of cricket olfactory sensory neurons will be essential. Equally critical will be future efforts to quantify natural bat odor composition, limonene release rates, ambient exposure concentrations, and odor-plume dynamics, which together will inform ecologically valid stimulus design in controlled assays.”

Lines 515–522: “For each antenna, a hexane control was first presented to establish baseline antennal activity. For the initial screening, limonene, undecane, pentadecane, and hexadecane were diluted to 10% (v/v) in hexane and delivered individually in a randomized order. To assess dose-dependent responses, limonene was further tested at five concentrations (0.001%, 0.01%, 0.1%, 1%, and 10%, v/v in n-hexane), following the concentration gradient used in a previous study (Tang et al., 2024). Following the initial hexane control, the five limonene concentrations were tested in a randomized order across trials. Each stimulus lasted 0.5 s, with an inter-stimulus interval of 1 min to allow full recovery of antennal responses.”

Lines 556–558: “Our assays were designed to test physiological detectability and functional sufficiency, rather than to establish concentration thresholds or mimic natural emissions exactly.”

(3) Figures 1C and D should compare the GC-EAD response of *L. equestris* to the odor of bat body and the odor of bat nasal secretions. It should not be compared with the air control group. Figure 1D has the same problem.

Thank you for this suggestion. We understand your point that comparing GC-EAD responses between bat body odor and snout secretions would be a more direct way to identify the anatomical source of active compounds.

However, we would like to explain why we did not include this comparison in the current study.

First, the purpose of Figures 1C and 1D (i.e., Figure 2C and 2D in the revised manuscript) was to answer a more fundamental question: whether bat body odor, as a whole, contains volatile compounds that are detectable by cricket antennae. Comparing with an odor-free air control was therefore the appropriate first step. It established the basic phenomenon of olfactory detection before we moved on to source attribution.

Second, we did conduct chemical profiling of snout secretions, hair, and faeces using HS-SPME-GC-MS (presented in Figure 3). These analyses showed that limonene was consistently present in hair and snout secretions, but absent from faeces and blanks. This allowed us to identify snout secretions as the most likely source of limonene, without requiring GC-EAD recordings from secretion samples themselves.

Third, we did not perform GC-EAD on snout secretions for practical reasons. The secretion samples were collected in very small amounts. They were almost entirely consumed during the HS-SPME-GC-MS chemical analyses, leaving insufficient material for additional GC-EAD testing.

We agree with you that directly comparing GC-EAD responses to snout secretions versus whole-body odor would be an excellent experiment. It would further strengthen the source attribution and provide more direct evidence. We have noted this as a valuable direction for future studies in the revised Discussion.

We hope this clarifies our rationale. Thank you again for your thoughtful suggestion.

**Recommendations for the authors:**

**Reviewer #1 (Recommendations for the authors):**

(1) I would suggest moving Supplementary Figure 1 into the main figures. It contains important information about the predator-prey relationship and the ecological relevance of *L. equestris*, so it seems too central to be placed only in the supplement.

We agree with your suggestion. The predator–prey relationship and the ecological relevance of *L. equestris* are indeed central to the biological context of this study. We have therefore moved the original Supplementary Figure 1 into the main text as Figure 1. We have renumbered the remaining figures accordingly, revised the figure legends, and updated the Results text to better highlight this ecological context (revised manuscript, Figure 1 legend, lines 856–864).

(2) Lines 134 to 136: Only one representative EAD trace is shown. I suggest showing all five traces, either in the main figure or as a supplementary figure, to better illustrate the reproducibility of the antennal responses across individuals.

We agree. To better illustrate reproducibility across individuals, we have added EAD traces from all five tested crickets as Supplementary Figure 1. The figure legend now describes the sample sizes for both the bat odor treatment and the odor-free control (revised manuscript, Supplementary Figure 1 legend, lines 900–906).

(3) Lines 144 to 153: It would be helpful if the authors reported which VOC collections contained limonene and in what amounts. Showing individual-level data for the bat odor samples, rather than only pooled or summarized profiles, would strengthen the conclusion that limonene is consistently associated with *S. kuhlii* body odor.

We agree. To better show the consistency of limonene detection across individuals, we have added Figure 3B, which displays an individual-level terpenoid profile. Each stacked bar represents one VOC collection, and the limonene segment indicates its presence and relative contribution. We have also revised the Results section to state explicitly that limonene was detected in hair and snout secretions but absent from feces (lines 144–149 in the revised manuscript).

Lines 144–149: “To identify the biological sources of bat body odor, we analyzed VOCs from hair, faeces, and snout (pararhinal gland) secretions of nine bats using headspace solid–phase microextraction coupled with gas chromatography–mass spectrometry (HS–SPME–GC–MS). Snout secretions and hair shared similar hydrocarbon-rich VOC profiles, whereas faecal volatiles were distinct (Figure 3A). Individual-level terpenoid profiles further showed that limonene was detected in hair and snout secretion VOC collections but was absent from faeces (Figure 3B and Supplementary Table 2).”

(4) Figure 2A: I recommend adding representative chromatograms or VOC traces for feces, hair, and snout secretions. This would make the source comparison more transparent and would help readers assess the underlying chemical profiles behind the heatmap.

We agree that representative chromatograms would make the source comparison more transparent. However, due to the analytical workflow, individual chromatograms were not retained in the dataset we received. As an alternative, we added Figure 3B, which shows individual-level terpenoid profiles. This allows readers to assess which samples contained limonene and how its relative contribution varied among sample types and individuals. In addition, we have provided the NIST retention index, quantitative ion, qualitative ion, and molecular weight for each terpenoid compound in Supplementary Table 2.

(5) The chemical identification of the key compounds would benefit from more detail. The authors state that compound identities were confirmed by matching retention times and mass spectra to authentic standards, including a mixed standard injection. It would be useful to provide the retention times, match and reverse-match scores, blank traces, and, if available, sample-plus-standard co-injection data showing peak augmentation without the appearance of new peaks. For (−)-limonene specifically, the enantiomeric assignment would require an enantioselective method, such as chiral gas chromatography, unless this was already performed and not described.

We fully agree that comprehensive identification evidence is important for transparency and reproducibility.

Regarding the identification data: we have already confirmed compound identities by matching retention times and mass spectra to authentic standards, including a mixed standard injection. In the revised version, we will deposit the raw chromatographic data and identification details (retention times, match scores, and blank traces) in Figshare as supporting information.

Regarding co-injection: we acknowledge that sample-plus-standard co-injection would provide even stronger confirmation. However, our bat odor samples were difficult to obtain and were almost entirely consumed during the GC–EAD and GC–MS analyses. We therefore could not perform additional co-injection validation. We have noted this limitation in the revised Materials and Methods (revised manuscript, lines 463–464).

Regarding the enantiomer assignment of limonene: you are correct that determining the specific enantiomer requires a chiral GC column, which was not available in this study. To avoid overinterpretation, we have replaced “(-)-limonene” with “limonene” throughout the manuscript.

**Reviewer #2 (Recommendations for the authors):**

(1) This is a typical study in the field of chemical ecology. As long as the source of the active substances is determined and the biological activity has been detected, this research is complete, so Figure 2 seems to be unnecessary.

We agree that identifying the source of the active compound and confirming its biological activity are central to this study. However, we believe Figure 2 serves an important purpose. Bat body odor could originate from multiple sources, i.e., hair, faeces, or snout secretions, and comparing VOC profiles across these sources helps us determine which source most likely contributes to the odor cues detected by crickets. This is especially important for limonene, because it is a plant-associated terpenoid and not a typical animal-derived volatile, as the reviewer #2 pointed out. Our analysis showed that hair and snout secretions shared similar VOC profiles, while faecal VOCs were distinct and lacked limonene. This supports snout secretions as the likely primary source. To make this purpose clearer, we have revised the Methods section to state that this analysis was conducted to investigate potential biological sources of bat body odor (revised manuscript, lines 494–495, 567–569).”

Line 494–495: “To identify the biological source of the characteristic body odor, we compared the VOC profiles from hair, faeces, and snout secretions.”

Line 567–569: “Principal component analysis (PCA) based on a binary (presence/absence) matrix was performed using the vegan package in R to compare profiles from hair, feces, snout secretions, and bat body odor.”

(2) Figure 3B seems to be incorrect. The significance of n-hexane and 1% limonene is ***P < 0.001, while for 10% limonene, why is it only two stars, **P < 0.01? Please check it.

We have rechecked the statistical analysis and confirmed that the original annotation was correct. The significance levels in original Figure 3B (Figure 4B in the revised manuscript) were based on Bonferroni-corrected paired t-tests comparing each limonene concentration with the n-hexane control. The adjusted P value was 0.0006 for 1% limonene (*P* < 0.001) and 0.00485 for 10% limonene (*P* < 0.01). The higher adjusted P value for 10% limonene reflects greater among-individual variation at this concentration, which may be due to differential sensitivity of individual antennae at higher doses. We have now added the exact adjusted P values in the revised manuscript, lines 163–167.

Lines 163–167: “EAG responses to limonene were concentration-dependent (repeated-measures ANOVA, F(5, 25) = 24.95, P < 0.001, η^2^p = 0.83; Figure 4B), with both 1% and 10% limonene solutions eliciting significantly stronger responses than the hexane control (Bonferroni-corrected paired t-tests, 1%: t(5) = 10.77, P < 0.001, Hedges' g = 3.82; 10%: t(5) = 6.92, P = 0.005, Hedges' g = 2.46).”

Finally, we would like to express our sincere gratitude to the editors and the reviewers for the thoughtful feedback. Your comments have significantly improved the quality and clarity of our manuscript. We hope the revised version satisfactorily addresses all concerns raised.
